# On a New Criterion for the Regulation of Blood-letting

**Published:** 1848-02

**Authors:** 


### PART IV.—SELECTIONS.

1. *On a New Criterion for the Regulation of Blood-letting.* (From a Review of Polli's Researches on the Blood. Med. Chirurg. Rev., Oct. 1847.)

[This criterion is deduced from the author's former series of researches, and confirmed by practical application in the hospital. It is thus stated to be "*The period of coagulation of the blood observed at different intervals of time between the abstractions, and in different portions of the mass taken during one blood-letting;*" and is amplified as follows :]

"1. Every time a large abstraction of blood is practised so as to lead to lipothymia, *the last portion* of that removed always coagulates with *greatest promptitude*, whatever may have been the time occupied by the first portion in coagulating. 2. Whenever, on the contrary, upon a person suffering from sanguineous congestion of the nervous centres, asphyxia, apoplexy, &c., bleeding is practised, and, by its use, the vital functions are again set at liberty, the last portion of the blood so removed coagulates *much more slowly* than that which was first emitted. 3. That it suffices to interrupt in some manner the course of the blood in the vein, or to diminish, by means of a ligature applied to an extremity, the irradiation of the nervous power, in order to secure the speedy coagulation of blood, which, a short time after, owing to the obstacles being removed, may reacquire the power of remaining long without coagulation. 4. That in diseases decidedly of an inflammatory and grave character, during which for the safety of the patient repeated blood-letting is requisite, if, on the occasion of every venesection, the coagulation of the first and last portions drawn be examined, it will be found that at the beginning the coagulation of the latter portion takes place *subsequently* to the former, and continues to do so in an equal ratio to the development of the morbid process, until this reaches its height. From this point, however, as the disease commences declining, the coagulation of the blood of the latter portion *precedes* that of the former. 5. That in cases in which abstraction of blood has been desisted from for some days, when the *slow coagulation of the last portion* taken announced a continuance of the phlogistic increment and the tolerance of blood-letting, it has become necessary to resort anew to this therapeutical agent, which can in no case be laid aside with the ready cure of the patient, unless the latter portion of the blood manifest an opposite disposition to that now pointed out. 6. That in opposite cases, in which the abstraction of blood is persisted in *notwithstanding its rapid coagulation* after all the venesections and during the two extreme periods of the same one, it has to be speedily renounced in consequence of the symptoms of intolerance which manifest themselves; and in those few unfortunate cases in which blood-letting is obstinately persevered in under the guidance of fallacious symptoms, vital exhaustion cuts short the career of the patient much more rapidly than would have done the course of the disease.

"It results, then, from these observations, that the maintenance of the fluid state of the blood, comparing one bleeding with another, or different periods of the same bleeding, is a measure of the vital energy proper to the individual, and of that brought into play by the morbid process; and that hence may be determined tolerance and indication of blood-letting; as on the other hand a prompt coagulation of the blood announces diminution of vital energy, or its exhaustion by the pathological action; and in every case that the power of governing the phlogistic or morbid vital movements is lowered."

[The criterion is of easy application, the first and last portions of blood drawn being separately collected in glass vessels, and placed at rest beyond the influence of disturbing causes before adverted to. As the difference of time employed by the blood in coagulating depends both upon the condition of the individual and the amount of blood drawn, the criterion in question may not only serve as a guide in judging of the propriety of bleeding in a certain contingency, but may determine the exact quantity to be drawn, and the period of its repetition.]

"Let an individual be bled to faintness, and you will always have the last portion of the blood rapidly coagulated, and consequently deprived of buffiness. Receive the blood into six, eight, or ten small recipients, of a similar form and nature, and the coagulation in the first will be in exact relation with the disposition of the fibrine to maintain itself in the liquid form proportionately to the particular physiological or morbid state of the organism; while in the last, such disposition will become gradually paralyzed and almost destroyed, from the gradually increasing effect of the abstraction itself. By contemplating this phenomenon, which is always a result bearing proportion to the two influences above alluded to, we are enabled to lay down a rule for in some cases practising abundant blood-letting at one time, in others practising it at intervals, or in small quantities ; or again simply interrupting its flow once or twice for some minutes during the abstraction, &c., accordingly as we may be desirous of obtaining a sudden subdual of the morbid exuberance of the vascular activity, or of securing a copious depletion without too great exhaustion of the strength, or the functional disturbance ensuing upon lipothymia, which may injuriously effect the regular course of some affections.

" From the different coagulation of the various portions of blood we may, moreover, as we have said, measure the *intensity of the inflammation* and the *tolerance of the individual* ; or, as others would express it, we may measure the morbid capacity and the amount of diathesis. There may, indeed, be a case in which the first portion of blood drawn indicates by its very slow coagulation a very high pathological condition, while the last portion announces in its rapid coagulation that the emission of blood has completely lowered the powers. This phenomenon may be dependent upon the existence of a very circumscribed, though a very intense affection, or upon exhaustion induced in an individual primarily possessed of very feeble powers of organic reaction ; and in such a case bleeding must be most reservedly employed, and frequently entirely rejected, for the reason that it is a less danger to leave the disease to proceed unchecked, than to have recource to means which remove it and the patient together. This difficult pathological circumstance, which a celebrated Italian physician justly compares to an *island of fire in a sea of ice*, is already known to practitioners as one which requires in the use of antiphlogistic measures great regard to be paid to the failure of the general strength. But, unfortunately, it has not always been easy to establish its diagnosis in time, or before unadvisably energetic therapeutical procedures have been put into force. But the criterion I propose informs us of these two opposite conditions co-existing in the same individuals, and measures their degree with a facility and security that no method of investigation hitherto recommended in these difficult cases can boast of."

[To the objection that the criterion only comes into operation after the abstraction of blood, Dr. Polli observes that in ordinary inflammatory diseases, the repetition of the bloodletting is the point to be inquired into; and that, even in those rare cases in which the diagnosis is very obscure, and in which a first bleeding might prove the cause of safety or of death, no harm whatever, and much good, would result from a very small *exploratory* venesection, and made in the view of obtaining the desired information. Such, consisting of one or two ounces, received in two separate vessels, should be instituted in all obscure cases of this kind, before resorting to an ordinary venesection.] "Perhaps even those small bleedings should be practised in all diseases indistinctly as a means of exploring the condition of the blood, for the same reason that, since auscultation has been employed upon all patients, it has not unfrequently revealed latent morbid conditions, to which the attention of the practitioner might otherwise not have been called until a more remote and a too late period." [A small subtraction which can do no harm to the economy, will yet depict to us the true characters of the vital condition of the tissues, and of the amount of the exaltation of the vascular activity and nervous function. It often suffices for the discovery of those circumscribed and concealed phlegmasiæ, which, frequently not spreading to such organs as would furnish external symptoms of their existence, pursue their undermining course until they have reduced the viscera they affect to such a condition, that some acute contingency at last suddenly betrays their formidable character.]

"Although the preservation of its fluidity by the blood, or the more or less time it requires for coagulation, constitutes for us the most certain measure of the activity of the phlogistic force, this is however, only curable in its indications in proportion to the stability of the morbid process itself. The phlogosis may, during its course, spontaneously increase or diminish in intensity, accordingly as it extends to neighboring tissues, or is confined to those first invaded. So that the different resistance of the blood to coagulation, which in every case announces with a rare exactitude the present state, cannot be extended, except within certain limits, to the indication of that which is to follow; since this latter can only be the complex and simultaneous effect of the condition of the development of the pathological lesion, and of the modification which the blood-letting itself may have induced. Our criterion, as expressing the present state of the organism, and the impression which the bleeding has developed, furnishes indications which are available for about twelve hours after, and may continue to be so for a much longer period, even to the supervention of complete health, providing new morbid causes and accidental inflammation do not supervene and complicate the course of the disease. And of this we may assure ourselves by the repeated observation of the coagulation of blood taken at brief intervals; since the times employed in the coagulation of the blood taken at the successive abstractions will generally glide, whether these are diminishing or increasing, gradually into each other, sudden variations not being observable, save when exacerbations or irregular complications coincide."

[In corroboration of the above views, tables are furnished of twenty cases of inflammatory disease observed in the hospital, for the relief of which were collectively performed 147 venesections. Notes to each case reported exhibit the author's views of the amount of corroboration derivable from it. Some of these are highly interesting, but we have only space to notice some of the practical conclusions he arrives at.]

"The observations already made upon the indications the physician may draw from the observation of the coagulation of the blood, and the clinical cases adduced in confirmation and illustration of this criterion, clearly prove that its value rather lies in its ennabling us to fix a limit to the abstraction than in encouraging its continuation. And, in fact, if we are not deceived, the comparison of the coagulation of the first and last portions may, independently of the presence of all other symptoms, distinctly indicate whether the evacuation will tend to normalize the vital powers of the functions of the organism, at one time liberating them from oppressive congestions, and at another from the obstacles presented by the excessive and unbalanced action of the nerves, or whether it attacks them with all its impoverishing effects, and directly exhaust the forces necessary to the carrying on of life. Of the two indications which this sign offers the least is not only the most important, since its neglect almost amounts to a fatal result in the disease, but it is also the most attainable, or at least the best supported by facts. The cases referred to show that, if the coagulation takes place with a certain celerity, and this manifests itself repeatedly, and goes on increasing with the blood-letting, we cannot persist in the measure without losing the patient; while the patient hardly ever dies when it is suspended prior to the coagulation having acquired great rapidity. * * * *

"It is not necessary for the complete cure of an inflammation to continue the bleedings until the blood no longer gives any buffiness; while it is absolutely necessary to cease the omission when the blood coagulates more rapidly than in the normal state. The production of buffiness of blood of equal coagulability, as shown in the former series, is always rendered more easy and in larger quantity after a certain number of bleedings than at first, in consequence of the diminished density which the blood acquires, which naturally always much diminishes the phlogistic expression, and the consequent indication for bleeding drawn from the crust that covers the blood after a certain number of emissions. The crust or buffiness, in fact not being *essentially* produced by an increase of fibrine, by a diminution of red globules, or by an attenuation of the serum, but arising from a certain slowness of the coagulation, (of that faculty by which, in certain morbid conditions of the organism, and especially under the influence of the phlogistic process, the fibrine has acquired the power of maintaining itself in a state of fluidity for a period always much longer than in the normal state,) it may at once disappear by the operation of whatever modifies that slowness. When the phlegmasia is subdued, and the morbid reactions give way to healthy movements, the blood will then undergo coagulation in a period of time that does not permit the appearance of the buffiness. It happens not unfrequently that if, for some reason independently of a reproduction of the phlegmasia, we draw blood during the advanced convalescence of a severe inflammation, in the treatment of which bleeding has been suspended, while the blood was yet covered with a firm phlogistic crust, it will now be found to present no trace whatever of this. A patient may have blood in circulation which if drawn would furnish a buffy crust, and who will yet be perfectly cured without blood-letting. This change in the blood within the vessels, without profuse crises inducing the belief that the morbid matters supposed to be indicated by the buffiness had been evacuated by other channels, frequently excited the surprise of the ancients; but faithful to observation, they had nevertheless laid down as a canon "*Ob solam crustan inflammatoriam venæsectio repetenda non est.*" (Quarin, *Met. Med. Inflam.*, p. 70.)—*Rankin's Abstract, Jan.* 1848.

